# Bilateral Wünderlich Syndrome Caused by Spontaneous Rupture of Renal Angiomyolipomas

**DOI:** 10.1155/2015/316956

**Published:** 2015-02-22

**Authors:** Stanislav Sotošek, Dean Markić, Josip Španjol, Kristian Krpina, Siniša Knežević, Anton Maričić

**Affiliations:** ^1^Department of Urology, University Hospital Rijeka, Tome Strižica 3, 51 000 Rijeka, Croatia; ^2^Department of Radiology, University Hospital Rijeka, Tome Strižica 3, 51 000 Rijeka, Croatia

## Abstract

Wünderlich syndrome (WS) is a urological emergency characterized by retroperitoneal hemorrhage. In most cases, bleeding occurs from a renal angiomyolipoma (AML) and may be the first manifestation of the disease. We report a female patient with bilateral WS due to the metachronous rupture of renal AMLs. Because the patient was stable and the tumor was not malignant, treatment was conservative. Follow-up revealed the full recovery of kidney function and the resolution of the hematoma.

## 1. Introduction

Wünderlich syndrome (WS) is a spontaneous, nontraumatic retroperitoneal hemorrhage associated with underlying renal pathology [1–3]. One of the most common causes of WS is the spontaneous rupture of a benign renal tumor, such as an angiomyolipoma (AML) [4]. It can manifest symptoms that range from being mild to being life threatening. We report a patient with bilateral WS due to the rupture of renal AMLs.

## 2. Case Report

**Figure 1:**
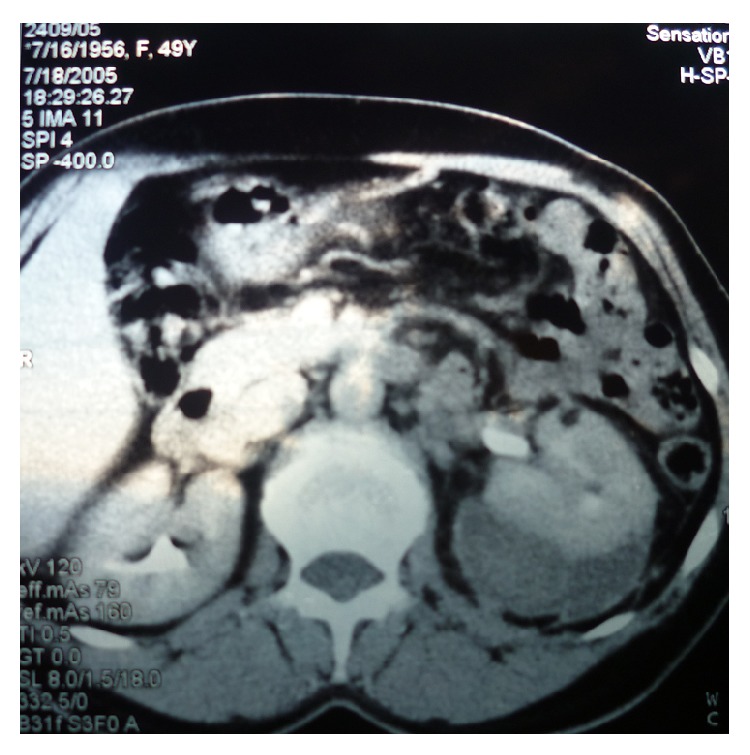
CT scan revealed a perirenal hematoma around the left kidney with an angiomyolipoma in the ventral part of the kidney.

**Figure 2:**
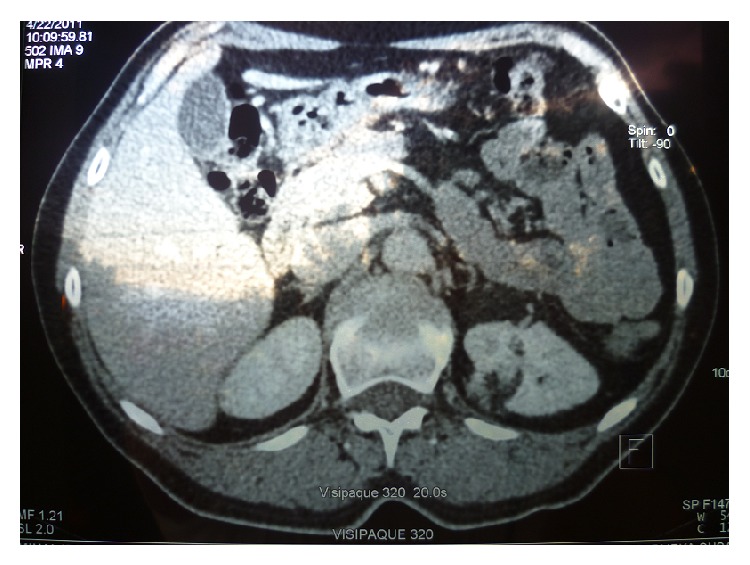
Six months after the spontaneous rupture of the angiomyolipoma (AML) on the left kidney, control CT showed the complete regression of the retroperitoneal hematoma and a well-delineated AML.

**Figure 3:**
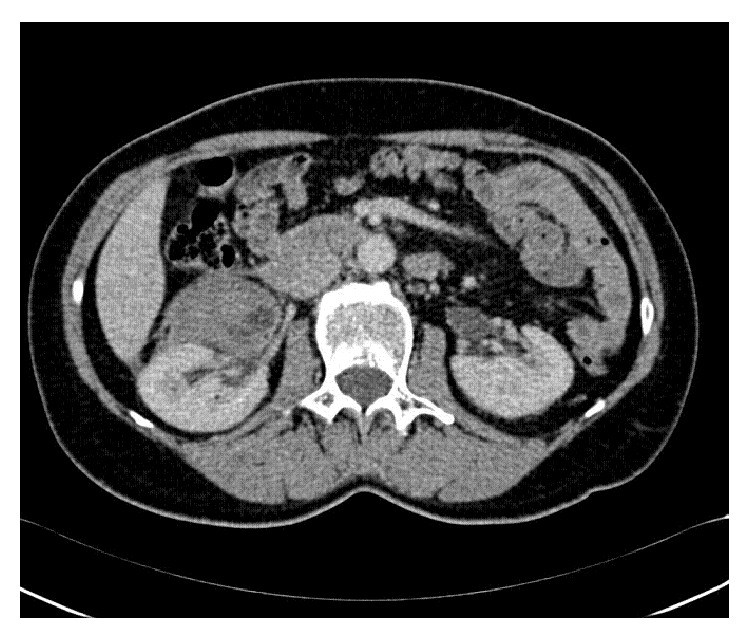
CT scan revealed a right perirenal hematoma after the rupture of the renal angiomyolipoma.

**Figure 4:**
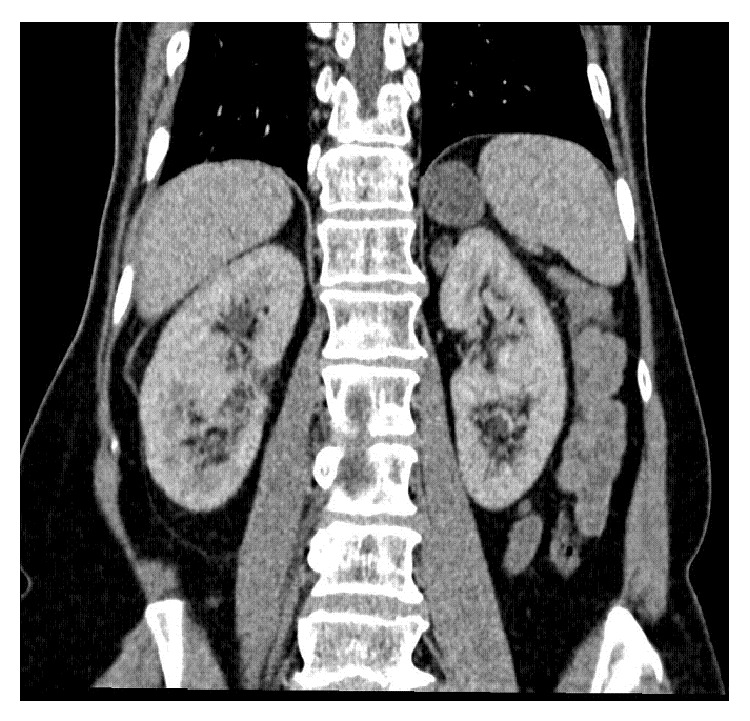
Six months after the rupture of the right renal angiomyolipoma, the complete resolution of the hematoma was observed, with normal renal parenchyma. The enlargement of the left adrenal gland (adenoma) was also observed.

A 49-year-old Caucasian female presented to the emergency department with abrupt-onset pain in the left flank, radiating to the left inguinal region. She had no concomitant diseases and no known history of trauma, anticoagulation, or bleeding diathesis. Physical examination revealed a mildly obese female with severe tenderness in the left flank and left upper quadrant. The patient was afebrile with normal blood pressure and without tachycardia. The patient's hemoglobin was 136 g/L, creatinine was 68 *μ*mol/L, and the estimated glomerular filtration rate was 88 mL/min. Urine sediment was normal. Abdominal ultrasound examination revealed a large retroperitoneal mass around the left kidney with heterogeneous echogenicity of the lower pole of the left kidney. For further evaluation, computed tomography (CT) scan of the abdomen and pelvis with intravenous contrast was performed, revealing a large left perinephric hematoma that distended the renal fascia and displaced the peritoneal and retroperitoneal structures. The estimated size of the hematoma was 15 cm in the cephalocaudal, 4 cm in the anteroposterior, and 7 cm in the transverse dimensions. In addition, a hypodense mass without calcification was detected in the lower pole of the left kidney that was poorly marginated from the perinephric hematoma (Figure 1). These findings were highly suggestive of the rupture of the angiomyolipoma of the left kidney with massive retroperitoneal hemorrhage, a condition known as WS. Because the patient was hemodynamically stable without significant changes in the hemoglobin level, conservative treatment was chosen, including the monitoring of vital signs (blood pressure and pulse), blood testing (hemoglobin and creatinine levels), and repeated ultrasound scans. She was initially administered intravenous fluids, after which she was placed on an oral diet and prescribed a broad-spectrum antibiotic. The patient remained stable throughout the hospital stay and was discharged ten days after admission. Follow-up examinations (including assessments of the hemoglobin and creatinine levels, ultrasound of the kidney, and CT scan) were performed 3 and 6 months after the initial presentation, revealing the complete morphological recovery of the left kidney with complete resolution of the perinephric hematoma (Figure 2). After five years, a similar clinical scenario was observed, but on the right side (Figure 3). The same diagnostic pathway with conservative management was performed with the complete resolution of the hematoma (Figure 4). Now, 10 years later, the patient has normal renal function without retroperitoneal hematoma. On ultrasound, both kidneys appear normal with two AMLs (3 cm on the left and 4 cm on the right sides). Because the renal AMLs are bilateral and the patient has experienced episodes of bleeding on both sides, she is educated about the possibility of repeated bleeding. She has also been informed to report immediately in the case of sudden abdominal pain. At this time, our patient has refused any proposed surgical procedures (nephron-sparing surgery).

## 3. Discussion

WS was first described in 1856 by the German physician Carl Reinhold August Wünderlich [1]. The clinical picture of Lenk's triad, including acute flank pain, abdominal tenderness, and signs of internal bleeding, is usually found in association with this syndrome. It can be caused by multiple etiologies, such as benign and malignant renal tumors, vascular lesions (polyarteritis nodosa), renal infections, undiagnosed hematological conditions, less common renal cysts, blood dyscrasias, or anticoagulant therapy [2, 3]. The most frequent cause of spontaneous retroperitoneal hemorrhage is renal angiomyolipoma [4, 5]. Eighty percent of AMLs are sporadic, typically occurring in middle-aged women, while the remaining 20% are associated with tuberous sclerosis [6, 7]. AML size is directly correlated with the risk of spontaneous rupture.

The best imaging technique for diagnosing WS is CT computed tomography [2]. This is also the method of choice for the demonstration of perirenal hemorrhage [4]. A confident diagnosis of AML can be made using CT to demonstrate the fat content of the lesion. Other renal tumors may also show a high fat content; however, a renal cortical mass composed predominantly of fat (less than −20 HU) can be diagnosed as AML [8]. In addition to CT scan, renal selective angiography can be performed, which can also be used for the embolization of bleeding vessels.

Once a patient is diagnosed with spontaneous perirenal hemorrhage due to AML, the proper treatment choice depends on the patient's clinical status, laboratory findings, and degree of kidney rupture and the size of the retroperitoneal mass. Many authors have argued that WS can be treated conservatively if a hemorrhage is self-limiting and a patient is responsive to fluid resuscitation [2, 7]. Conservative management is the most acceptable option for stable patients, unless malignant pathology can be demonstrated [8]. For patients who are clinically unstable, surgery is the first option chosen. For most of these cases, nephrectomy is performed. Unfortunately, nephrectomy carries a high incidence of morbidity due to the loss of renal function and complications associated with hemodialysis. Due to the benign nature of AML, patients can undergo nephron-sparing surgery, which can be performed either by open surgery or by a minimally invasive procedure [9]. Selective arterial embolization, which is also a nephron-sparing and minimally invasive procedure, can be utilized in such cases to reveal vascular lesions that are not visible on CT scan.

A bilateral presentation of WS is very rare. Agarwal et al. presented a patient with bilateral retroperitoneal hemorrhage caused by polyarteritis nodosa and treated by embolization of the bleeding artery [10].

Our case of metachronous spontaneous rupture of bilateral AMLs of the kidneys is interesting because it demonstrates that even relatively small renal AMLs can spontaneously rupture. The diagnosis of our patient was performed by abdominal CT, not only to confirm the retroperitoneal hemorrhage but also to reveal the cause of the retroperitoneal bleeding. The findings of our patient support the conservative treatment of patients who are stable when the presence of a malignant tumor has been excluded. It will be interesting to observe the future behaviors of these lesions, for example, whether the hemorrhage will occur again. If repeated episodes of rupture occur, nephron-sparing surgery or embolization can be recommended during the remission phase.
